# Molecular structure studies of (1*S*,2*S*)-2-benzyl-2,3-dihydro-2-(1H-inden-2-yl)-1H-inden-1-ol

**DOI:** 10.1016/j.molstruc.2014.12.018

**Published:** 2015-03-05

**Authors:** Tao Zhang, Krzysztof Paluch, Gaia Scalabrino, Neil Frankish, Anne-Marie Healy, Helen Sheridan

**Affiliations:** a Trino Therapeutics Ltd, The Tower, Trinity Technology and Enterprise Campus, Dublin 2, Ireland; b Novel Drug Discovery Group, School of Pharmacy and Pharmaceutical Sciences & Trinity Biomedical Sciences Institute, Trinity College, Dublin 2, Ireland; c Centre for Pharmaceutical Engineering Science, Bradford School of Pharmacy, Faculty of Life Sciences, University of Bradford, Richmond Road, Bradford BD7 1DP, UK

**Keywords:** (1*S*,2*S*)-2-Benzyl-2,3-dihydro-2-(1H-inden-2-yl)-1H-inden-1-ol, Single enantiomer, Chemical separation, NMR, X-ray, XRPD

## Abstract

The single enantiomer (1*S*,2*S*)-2-benzyl-2,3-dihydro-2-(1H-inden-2-yl)-1H-inden-1-ol (**2**), has recently been synthesized and isolated from its corresponding diastereoisomer (**1**). The molecular and crystal structures of this novel compound have been fully analyzed. The relative and absolute configurations have been determined by using a combination of analytical tools including X-ray crystallography, X-ray Powder Diffraction (XRPD) analysis and Nuclear Magnetic Resonance (NMR) spectroscopy.

## Highlights

• Chemical separation is used for racemate resolution.
• NMR analyses are used for single enantiomer structure education.
• Intermolecular interactions are observed by X-ray study.

## Introduction

The indane moiety is present in many bioactive pharmaceutical products including the non-steroidal anti-inflammatory indane sulindac (Clinoril, Merck) [1], [2], [3] and the protease inhibitor indinavir (Crixivan, Merck) used as a component of highly active antiretroviral therapy (HAART) [4], [5]. As part of our ongoing drug discovery programme, we have synthesized a variety of indane molecules using different synthetic approaches [6], [7], [8], [9], [10], [11], [12]. Many of these novel molecules have been shown to possess smooth muscle relaxation properties as well as mediator release inhibition [6], [7], [8]. More recently, a series of anti-inflammatory dimeric indane compounds with potential therapeutic value have been synthesized and characterized in our group [10], [11], [13], [14].

**Scheme 1:**
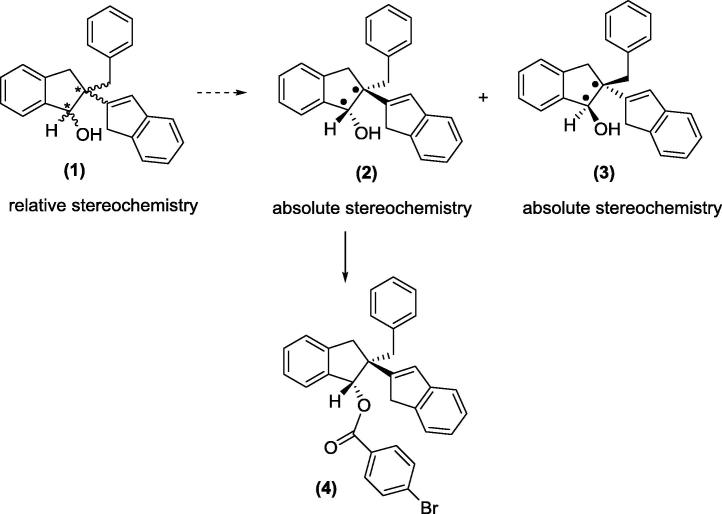
Formation of enantiomer (**2**) derivative, compound (**4**).

**Scheme 2:**
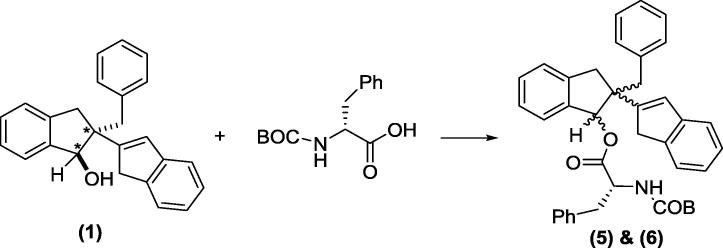
Chemical separation of enantiomers (**2**) and (**3**): formations of *N*-BOC D-phenylalanine derivative of compounds (**5**) and (**6**).

**Scheme 3:**
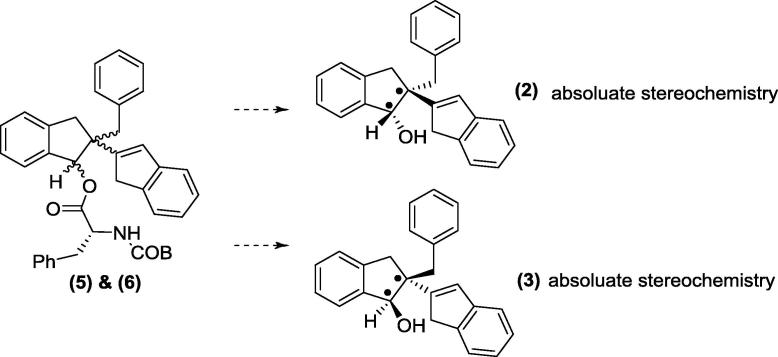
Chemical separation of enantiomers (**2**) and (**3**): isolation of enantiomers (**2**) and (**3**).

This work has been challenged by the need to synthesis and resolve single enantiomeric forms of bioactive compounds. In the current study we report the separation of the single enantiomers (**2**) and (**3**) of a potent anti-inflammatory diastereoisomeric mixture (**1**) [10]. The molecular structure of enantiomer (**2**) was investigated using its derivative, compound (**4**) (Scheme 1) and the relative & absolute configurations of enantiomer (**2**) were also subsequently determined. In this paper, we report the synthetic chemistry including separation of enantiomers (Scheme 2) and indanol esterification (Scheme 3). Single crystal X-ray crystallographic analysis, X-ray Powder Diffraction (XRPD) and Nuclear Magnetic Resonance (NMR) analysis were performed on compound (**4**).

## Experimental

### Chemical separation of constituent enantiomers (**2**) & (**3**) from diastereoisomer (**1**)

Diastereoisomer (**1**), 2-benzyl-2,3-dihydro-1H,1′H-[2,2′-biinden]-1-ol, was prepared as detailed by Sheridan et al. [10]. This single diastereoisomer was derivatized with *N*-BOC D-phenylalanine (Scheme 2); the diastereoisomers (**5**) and (**6**) formed from this reaction were chromatographically separated and subsequently the phenylalanine group was removed by hydrolysis to give two enantiomers (**2**) and (**3**) (Scheme 3).

#### Preparation of *N*-BOC D-phenylalanine derivative of (**5**) & (**6**)

To a stirred solution of compound (**1**) (0.41 g, 1.201 mmol) and *N*-BOC D-phenylalanine (0.40 g, 1.509 mmol) in acetonitrile (8 mL) was added pyridine (0.12 mL, 1.5 mmol). The mixture was stirred at room temperature and a solution of *N*,*N′*-dicyclohexylcarbodiimide (DCC) (1.5 mmol, 0.309 g) and 4-dimethylaminopyridine (DMAP) (0.02 g, 10 mol%,) in acetonitrile (2 mL) was added drop-wise. After a short time, a white precipitate was seen. The reaction was heated to 50–60 °C for 2 h. On cooling, the reaction mixture was filtered and the dicyclohexylurea precipitate was washed well with acetonitrile. The acetonitrile was evaporated *in vacuo* and the residue was taken up in ethyl acetate (20 mL). The ethyl acetate extract was washed with 1 N sulfuric acid (20 mL), saturated sodium bicarbonate solution (20 mL), brine (20 mL), dried over anhydrous magnesium sulphate and evaporated to give a yellow oil which contained two diastereoisomers (**5**) and (**6**) (TLC analysis: hexane:methyl *tert*-butyl ether (MTBE), 4:1). The diastereoisomers were separated by flash column chromatography (stationary phase: silica gel 230–400 mesh; mobile phase: 95:5, hexane:MTBE) to give diastereoisomer (**5**): 0.335 g (48%); diastereoisomer (**6**) 0.323 g (46%).

#### Hydrolysis of *N*-BOC D-phenylalanine derivative of diastereoisomer (**1**)

To a stirred solution of *N*-BOC D-phenylalanine derivative of diastereoisomer (**5**) or (**6**) (0.66 g, 1.128 mmol) in methanol (25 mL) was added potassium carbonate (0.17 g, 1.232 mmol). The reaction mixture was heated at reflux and monitored by TLC (hexane:MTBE, 80:20). After 2 h, no further starting material was seen. The methanol was removed *in vacuo* and the solid residue taken up in water and ethyl acetate. The layers were separated and the aqueous layer extracted with ethyl acetate (3 × 25 mL). The combined organic layers were washed with water (3 × 50 mL), brine (50 mL), dried over anhydrous magnesium sulphate and evaporated. The crude product was purified by flash column chromatograph (stationary phase: silica gel 230–400 mesh; mobile phase: hexane:MTBE, 90:10) as eluent to give 0.34 g (90%) of the product as off-white solid.

##### Enantiomer (**2**)

**M.P.** 59–64 °C; **[α]_D_**: −65.52 (7.67%, CHCl_3_); **δ_H_ (400** **MHz, CDCl_3_)**: 2.80 (1H, *d*, *J* = 13.40 Hz, CH_2_), 3.02 (1H, *d*, *J* = 15.56 Hz, CH_2_), 3.12 (1H, *d*, *J* = 15.56 Hz, CH_2_), 3.24 (1H, *d*, *J* = 13.40 Hz, CH_2_), 3.49 (1H, *d*, *J* = 22.60 Hz, CH_2_), 3.62 (1H, *d*, *J* = 22.60 Hz, CH_2_), 5.27 (1H, *s*, CHOH), 6.53 (1H, *s*, CH=C), 6.93 (2H, *s*, Ar–H), 7.20–7.30 (9H, 2 × *m*, Ar–H), 7.46–7.48 (2H, *m*, Ar–H); **δ_C_ (86.5 MHz, CDCl_3_)**: 2 × 38.0 (2× CH_2_), 39.6 (CH_2_), 55.4 (quat. C), 82.7 (CHOH), 120.0 (tert. C), 123.1 (tert. C), 123.5 (tert. C), 123.6 (tert. C), 124.5 (tert. C), 125.7 (tert. C), 125.9 (tert. C), 126.5 (tert. C), 2 × 127.4 (2 × tert. C), 127.8 (tert. C), 128.0 (tert. C), 2 × 129.7 (2 × tert. C), 137.9 (quat. C), 140.3 (quat. C), 142.5 (quat. C), 143.4 (quat. C), 144.3 (quat. C), 152.7 (quat. C).

##### Enantiomer (**3**)

**M.P.** 57–60 °C; **[α]_D_**: +64.72 (7.79%, CHCl_3_); **δ_H_ (400 MHz, CDCl_3_)**: 2.80 (1H, *d*, *J* = 13.40 Hz, CH_2_), 3.02 (1H, *d*, *J* = 15.56 Hz, CH_2_), 3.12 (1H, *d*, *J* = 15.56 Hz, CH_2_), 3.24 (1H, *d*, *J* = 13.40 Hz, CH_2_), 3.49 (1H, *d*, *J* = 22.60 Hz, CH_2_), 3.62 (1H, *d*, *J* = 22.60 Hz, CH_2_), 5.27 (1H, *s*, CHOH), 6.53 (1H, *s*, CH=C), 6.93 (2H, *s*, Ar–H), 7.20–7.30 (9H, 2 × *m*, Ar–H), 7.46–7.48 (2H, *m*, Ar–H); **δ_C_ (86.5 MHz, CDCl_3_)**: 2 × 38.0 (2× CH_2_), 39.6 (CH_2_), 55.4 (quat. C), 82.7 (CHOH), 120.0 (tert. C), 123.1 (tert. C), 123.5 (tert. C), 123.6 (tert. C), 124.5 (tert. C), 125.7 (tert. C), 125.9 (tert. C), 126.5 (tert. C), 2 × 127.4 (2 × tert. C), 127.8 (tert. C), 128.0 (tert. C), 2 × 129.7 (2 × tert. C), 137.9 (quat. C), 140.3 (quat. C), 142.5 (quat. C), 143.4 (quat. C), 144.3 (quat. C), 152.7 (quat. C).

### Derivatisation of enantiomer (**2**)

**Scheme 4:**
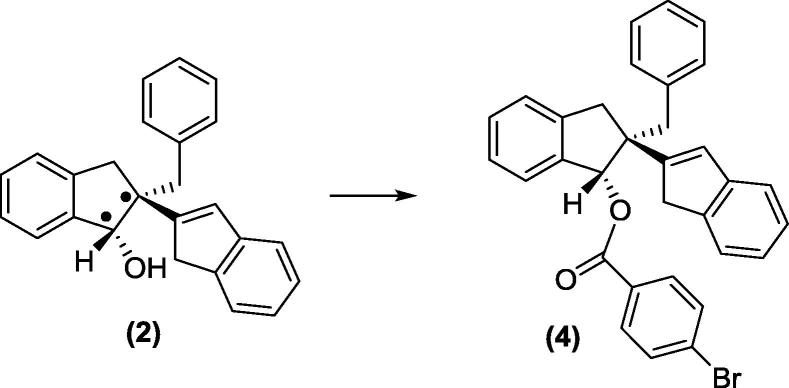
Formation of enantiomer derivative (**4**).

Enantiomer (**2**), (1*S*,2*S*)-2-benzyl-2,3-dihydro-1H,1′H-[2,2′-biinden]-1-ol, was derivatized with 4-bromobenzoic acid to give enantiomeric compound (**4**). All subsequent molecular structure analysis were performed on this derivative (Scheme 4).

To a stirred solution of enantiomer (**2**) (0.06 g, 0.177 mmol) and 4-bromobenzoic acid (0.05 g, 0.266 mmol) in dry dichloromethane (5 mL) was added 4-dimethylaminopyridine (0.03 g, 0.266 mmol), diisopropylethylamine (0.05 mL, 0.266 mmol) and 2,6-dichlorobenzoyl chloride (0.04 mL, 0.266 mmol) under an atmosphere of nitrogen. After an hour the reaction was quenched by the addition of saturated sodium bicarbonate solution (10 mL) and the product was extracted with diethyl ether (3 × 20 mL). The organic layers were combined and dried over anhydrous magnesium sulphate and concentrated *in vacuo.* The resulting oily residue was then purified by flash column chromatography (stationary phase: silica gel 230–400 mesh; mobile phase: 10:1, hexane:ethyl acetate,) to yield target ester product, (1*S*,2*S*)-2-benzyl-2,3-dihydro-2-(1H-inden-2-yl)-1H-inden-1-yl 4-bromobenzoate (**4**), as a pale yellow oil (0.08 g, 99%). Analytical data of this ester is shown below. Compound (**4**) was then crystallized from the mixture of acetonitrile and isopropanol (*v:v*, 1:1).

**M.P.** 156–158 °C; **HRMS (**+**Na**^+^**):** 543.0930*m*/*z*, required 543.0912*m*/*z*, C_32_H_25_O_2_BrNa; **δ_H_(600 MHz, CDCl_3_):** 3.17 (1H, *d*, *J* = 13.56 Hz, CH_2_), 3.19 (1H, *d*, *J* = 15.42 Hz, CH_2_), 3.27 (1H, *d*, *J* = 22.52 Hz, CH_2_), 3.366 (1H, *d*, *J* = 13.56 Hz, CH_2_), 3.371 (1H, *d*, *J* = 15.42 Hz, CH_2_), 3.41 (1H, *d*, *J* = 22.52 Hz, CH_2_), 6.57 (1H, *s*, CH=C), 6.64 (1H, *s*, CHOC=O), 6.94–6.95 (2H, *overlapping signals*, Ar–H), 7.15 (1H, *dt*, *J*_1_ = 1.23 Hz, *J*_2_ = 7.29 Hz, Ar–H), 7.15–7.19 (3H, *m*, Ar–H), 7.21–7.24 (2H, *m*, Ar–H), 7.27 (1H, *d*, *J* = 7.16 Hz, Ar–H), 7.31–7.32 (2H, *m*, Ar–H), 7.36 (1H, *overlapping d*, *J* = 7.60 Hz, Ar–H), 7.39 (1H, *overlapping d*, *J* = 7.42 Hz, Ar–H), 7.67 (2H, *overlapping d*, *J* = 8.68 Hz, Ar–H), 8.05 (2H, *overlapping d*, *J* = 8.52 Hz, Ar–H); **δ_C_(150 MHz, CDCl_3_):** 39.6 (CH_2_), 40.4 (CH_2_), 40.8 (CH_2_), 54.9 (quat. C), 83.8 (CHOC=O), 120.6 (tert. C), 123.5 (tert. C), 124.3 (tert. C), 124.6 (tert. C), 125.7 (tert. C), 126.31 (tert. C), 126.33 (tert. C), 126.9 (tert. C), 2 × 127.9 (2 × tert. C), 128.5 (quat. C), 129.08 (tert. C), 129.1 (quat. C), 129.5 (CH=C), 2 × 130.0 (2 × tert. C), 2 × 131.3 (2 × tert. C), 2 × 131.9 (2 × tert. C), 137.9 (quat. C), 140.4 (quat. C), 142.2 (quat. C), 142.7 (quat. C), 144.4 (quat. C), 151.6 (CH=C), 165.6 (C=OOAr).

### X-ray crystallography

**Fig. 1:**
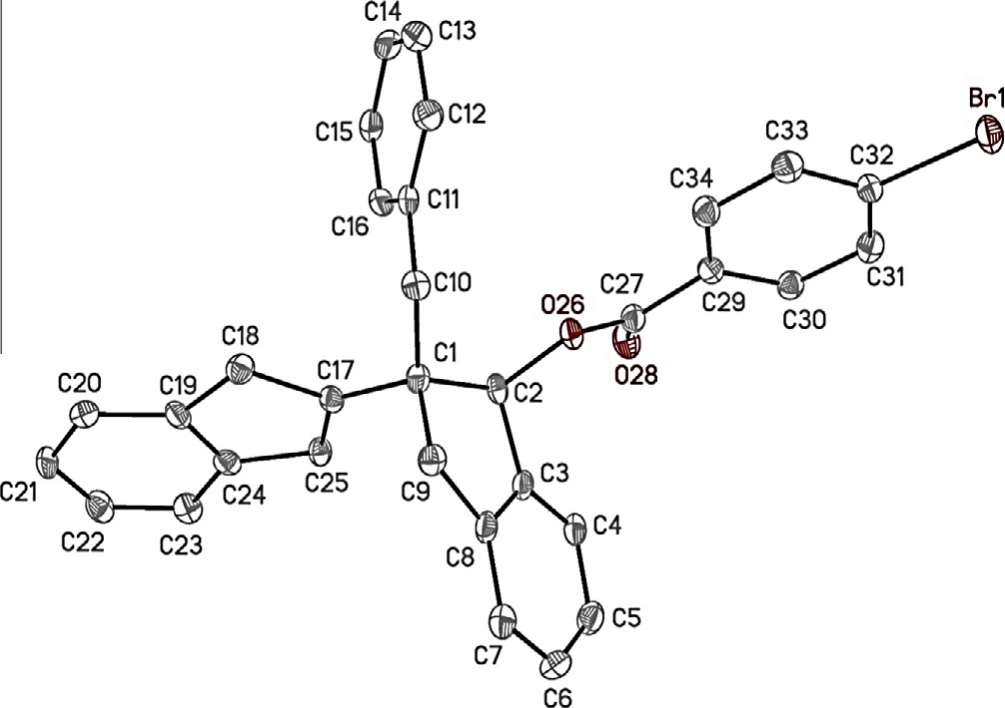
The molecule structure of the enantiomeric compound (**4**) with the atom numbering scheme. Displacement ellipsoids are shown at the 50% probability level. H atoms are omitted for clarity.

A single crystal X-ray analysis of compound (**4**) was carried out (Fig. 1). A specimen of C_32_H_25_BrO_2_, approximate dimensions 0.180 mm × 0.200 mm × 0.250 mm, was used for the X-ray crystallographic analysis. The X-ray intensity data were measured at 150(2) K using an Oxford Cryosystems Cobra low temperature device. A total of 3355 frames were collected. The total exposure time was 11.37 h. Cell parameters were obtained using CELL_NOW-2008/4 [15] giving a two component twin, with the second domain rotated from first domain by 179.7° about the reciprocal axis −0.080, 1.000, 0.020; and real axis −0.010, 1.000, 0.000. The frames were integrated with the Bruker SAINT software package using a narrow-frame algorithm. The integration of the data using a monoclinic unit cell yielded a total of 7391 reflections to a maximum *θ* angle of 67.14° (0.84 Å resolution). The final cell constants are based upon the refinement of the XYZ-centroids of 8691 reflections above 20 *σ*(*I*) with 10.25° < 2*θ* < 134.2°. Data were corrected for absorption effects using the multi-scan method TWINABS-2012/1. The calculated minimum and maximum transmission coefficients (based on crystal size) are 0.5217 and 0.7529. The structure was solved and refined using the Bruker SHELXTL-2014 Software Package. The largest peak in the final difference electron density synthesis was 0.724 e^−^/Å^3^ and the largest hole was −0.420 e^−^/Å^3^ with an RMS deviation of 0.079 e^−^/Å^3^. On the basis of the final model, the calculated density was 1.450 g/cm^3^ and F(000), 536 e^−^. The crystallographic data was analyzed using: Mercury 2.2 [16] and Ortep-3 [17].

#### Crystal data

Compound (**4**), C_32_H_25_BrO_2_, *M* = 521.43, Monoclinic, *a* = 13.7096(6), *b* = 6.0743(3), *c* = 14.8934(7) Å, *β* = 105.613(2)°, *U* = 1194.50(10) Å^3^, *T* = 146(2) K, space group P2_1_, *Z* = 2, μ (Cu Kα) = 2.562 mm^−1^, *ρ* = 1.450 Mg/cm^3^, 7391 reflections collected, 6935 unique, (*_R_*_int_ = 0.0750), *R* indices (all data) *^a^_R_*_1_ = 0.0409, *wR*2 [*I* > 2*σ*(*I*)] = 0.1069, *Gof* on *F*^2^ = 0.814, Flack = 0.010(14), BASF (twin ratio) = 0.43974. Solved as a rotational twin using CELL_NOW-2008/4.

### X-ray Powder Diffraction (XRPD)

The crystals of compound (**4**) was subsequently characterized by XRPD. XRPD analysis was conducted using a Rigaku Miniflex II Desktop X-ray diffractometer (Tokyo, Japan) with an Haskris cooling unit (Grove Village, IL, USA). The tube output voltage used was 30 kV and tube output current was 15 mA. A Cu-tube with Ni-filter suppressing Kβ radiation was used. Measurements were taken from 5 to 40 on the 2 theta scale at a step size of 0.05° per second in each case. Scans were performed at room temperature.

### Nuclear magnetic resonance

Bruker Avance DRX-600 Spectrometer was used for NMR studies. ^1^H and ^13^C NMR spectra were recorded at 600 MHz with chemical shifts expressed in parts per million (ppm or δ) down field from the standard and 150 MHz respectively. The number of scans was appropriate to generate good quality spectra for analysis. Both 1D and 2D NMR experiments were analysed with Bruker Topspin 2.1^™^ software.

## Results and discussion

### X-ray crystallographic analysis and short-range contacts network study

The absolute stereochemistry of for compound (**4**) was determined as *S*, *S* at C1 and C2 positions. The assignment was made from consideration of both the Flack parameter which was determined to be 0.010(14) and from the *a priori* knowledge of the stereochemistry of this ester former. The absolute configuration of the ester precursor, compound (**3**) was therefore believed to be *S*, *S.*

**Fig. 2:**
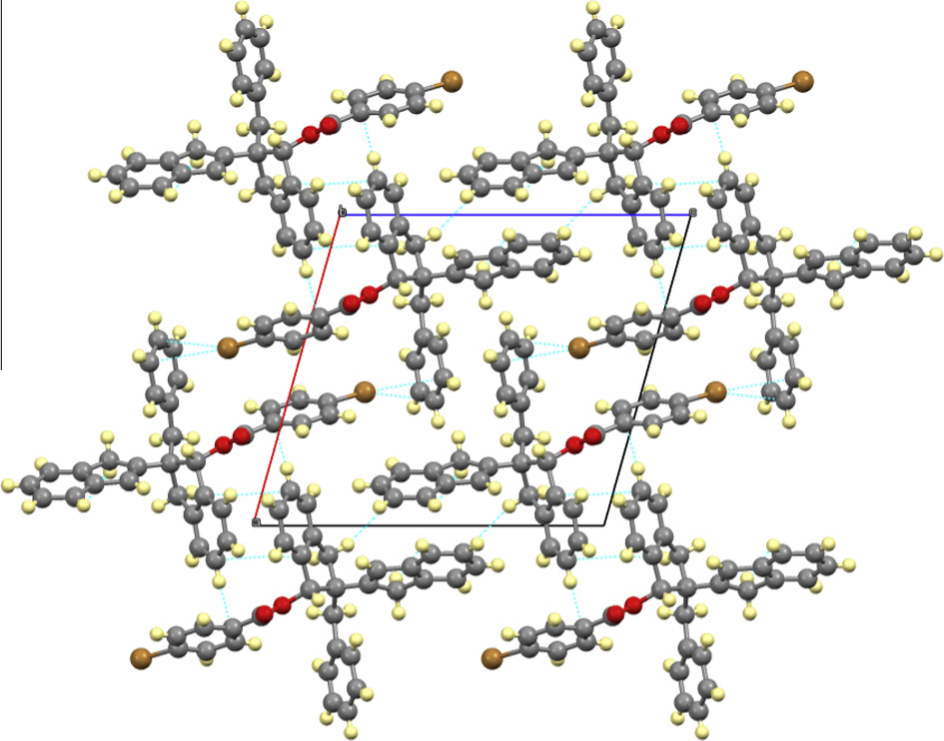
Short range contacts in crystal lattice of compound (**4**) along *b* axis.

In the crystal lattice of compound (**4**), there is no hydrogen bonds. All present short-range contacts are intermolecular (Table 1 and Fig. 2). The majority of the short-range contacts are formed between indene moieties of compound (**4**) in addition the benzene ring contacts with indene moiety and with bromine atom attached to the benzene ring of compound (**4**).

**Table 1:** List of short-range contacts in the crystal lattice of compound (**4**).

Atom 1	Atom 2	Length (Å)	Length-VdW (Å)	Symmetry
C23	H18b	2.883	−0.034	*x*, −1 + *y*, *z*
H23a	H18b	2.340	−0.117	*x*, −1 + *y*, *z*
C4	H9b	2.585	−0.307	*x*, −1 + *y*, *z*
H4a	H9b	2.169	−0.201	*x*, −1 + *y*, *z*
H6a	C34	2.821	−0.089	−*x*, −1/2 + *y*, −*z*
H4a	C6	2.911	−0.005	−*x*, −1/2 + *y*, −*z*
C22	H9a	2.858	−0.069	−*x*, −1/2 + *y*, 1 − *z*
C15	Br01	3.468	−0.081	1 − *x*, −1/2 + *y*, −*z*
C14	Br01	3.385	−0.172	1 − *x*, −1/2 + *y*, −*z*

**Fig. 3:**
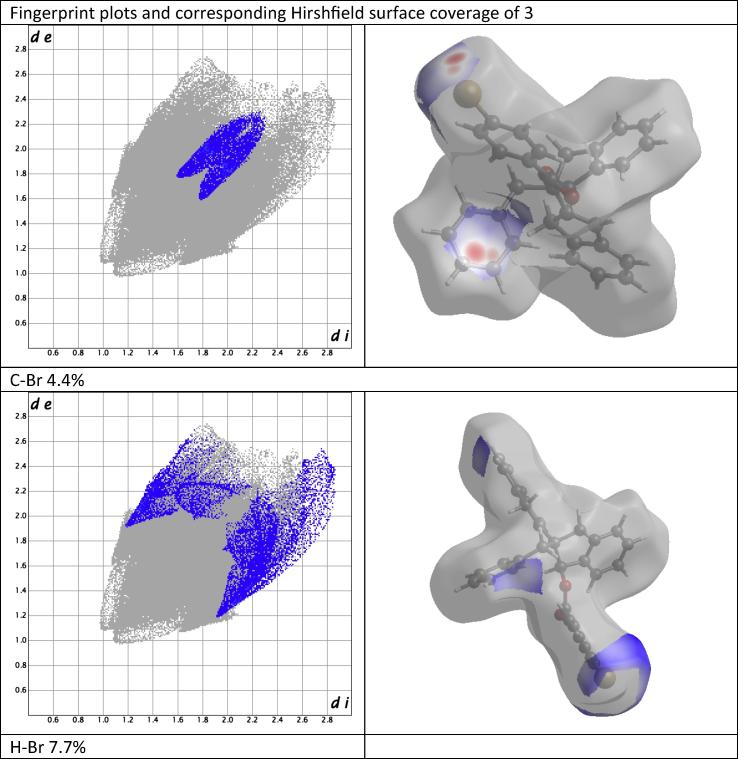

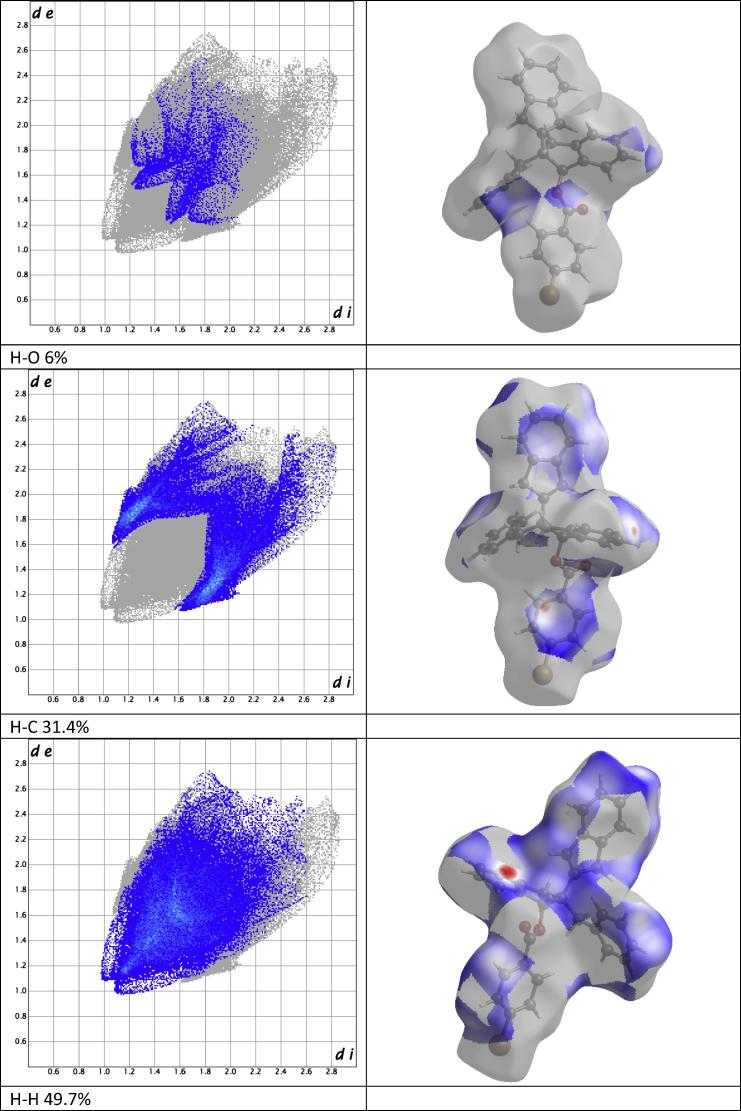
Fingerprint plots and corresponding Hirshfeld surface coverage of compound (**4**).

All short-range contacts in Table 1 were depicted in Hirshfeld surface (HS) analysis [18] as a red hotspots (Fig. 2) corresponding to *d*_norm_ reciprocal interactions [19], [20], [21]. Red^1^ hot spots indicate interactions stronger than Van der Waals interactions such as hydrogen bonds and/or weaker short-range contacts. HS analysis allows to calculate a percentage of involvement of inter- and intra-molecular atoms reciprocal interactions into overall coverage of HS which is generated on boundary of intermolecular interactions. HS may be converted to two-dimensional presentation of interactions called fingerprint plot (Fig. 3) [22]. C15–Br01 and C14–Br01 short-range contact corresponds only to 4.4% of overall HS and covers shorter de/di distance interactions from 1.6–1.8 Å up to 2.2–2.2 Å [23]. Even weaker than short-range interactions were depicted between hydrogens and bromine or oxygen atoms of carbonyl group, though they covered larger HS area. Respectively H–Br and H–O interactions covered 7.7% and 6.0% of HS. The largest input in HS coverage have inter- and intra-molecular interactions between hydrogens itself and hydrogens and carbons of compound (**4**). Respectively H–C and H–H interactions cover 31.4% and 49.7% of HS in Fig. 3. Apart they cover the largest HS area, taking into account that the shorter is distance between interacting atoms the stronger is its reciprocal interaction, H–C and H–H interactions form the strongest short range contacts respectively between C4–H9b and H6a–H9b both related to indene moiety of compound (**4**) structure.

### X-ray Powder Diffraction (XRPD)

**Fig. 4:**
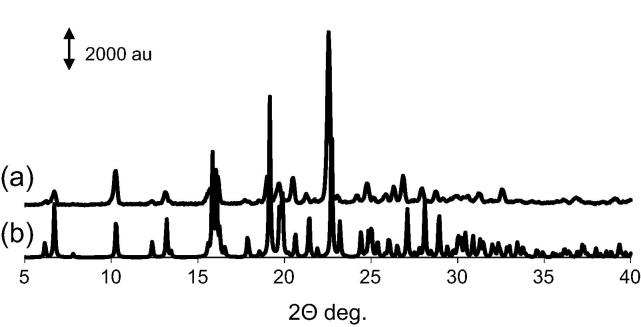
XPRD analysis of compound (**4**): (a) experimental XPRD pattern for compound (**4**); (b) the simulated XPRD pattern calculated from single-crystal structure of compound (**4**).

The theoretical XRPD pattern of compound (**4**) generated from the single crystal X-ray analysis at wavelength 1.54056 Å, is consistent with the experimental XRPD pattern of the bulk materials determined at ambient conditions (Fig. 4), which confirmed the structure of compound (**4**) determined by single crystal analysis is representative of the bulk material. The differences between the two patterns in relation to peaks intensity and broadening may be respectively related to crystal habit of the material, preferred orientation of crystallites in the sample holder and thermal anisotropic expansion of the lattice.

### Nuclear magnetic resonance

**Fig. 5:**
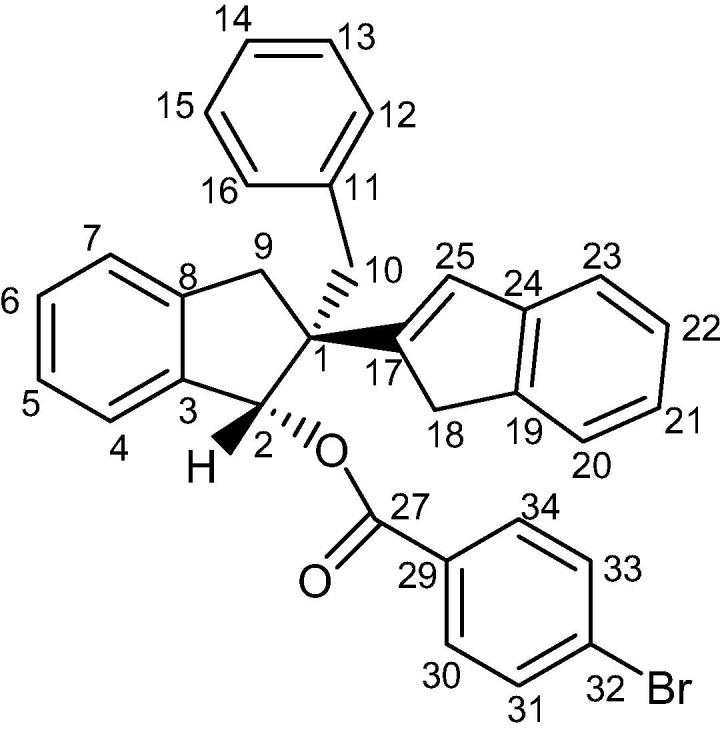
Chemical structure of compound (**4**).

The enantiomeric derivative (**4**) was also subjected to NMR analysis. A variety of 1D and 2D NMR spectroscopic methods were used to characterise the compound (Fig. 5). These include various 1D and 2D NMR techniques including ^1^H, ^13^C, Distortionless Enhancement of Polarisation Transfer (DEPT), 2D Proton–Proton Correlation Spectroscopy (H–H COSY), 2D Heteronuclear Single Quantum Coherence (HSQC), 2D Heteronuclear Multiple-Bond Correlation Spectroscopy (HMBC), 1D selective Total Correlation Spectroscopy (TOSY), 1D selective Nuclear Overhauser Enhancement Spectroscopy (NOSEY) and 2D NOSEY. As a result, all atoms of compound (**4**) were successfully assigned and the relative/absolute configuration was further confirmed (Table 2).

**Table 2:** ^1^H and ^13^C NMR Spectral Assignments of compound (**4**).

Atom number	^1^H chemical shift (ppm)	Pattern	Coupling constant (Hz)	^13^C chemical shift (ppm)
1	–	–	–	54.9
2	6.64	Singlet	–	83.8
3	–	–	–	140.4
4	7.39	Overlapping doublet	7.42	125.7
5	7.23	Overlapping signals	N/A	126.9
6	7.319	Overlapping signals	N/A	129.08
7	7.311	Doublet	4.09	124.6
8	–	–	–	142.2
9	3.371	Doublet	15.42	40.4
	3.19	Doublet	15.42	
10	3.17	Doublet	13.56	40.8
	3.366	Doublet		
11	–	–	–	137.9
12	6.94	Overlapping signals	–	130.0
13	7.178	Overlapping signals	–	127.9
14	7.18	Overlapping signals	–	126.31
15	7.178	Overlapping signals	–	127.9
16	6.94	Overlapping signals	–	130.0
17	–	–	–	151.6
18	3.27	Doublet	22.52	39.6
	3.41	Doublet	22.52	
19	–	–	–	142.7
20	7.36	Overlapping doublet	7.60	123.5
21	7.15	Double triplet	1.23, 7.29	124.3
22	7.24	Overlapping signals	–	126.33
23	7.27	Triplet	7.16	120.6
24	–	–	–	144.4
25	6.57	Singlet	–	129.5
27	–	–	–	165.6
29	–	–	–	129.1
30	8.05	Overlapping doublet	8.52	131.3
31	7.67	Overlapping doublet	8.68	131.9
32	–	–	–	128.5
33	7.67	Overlapping doublet	8.68	131.9
34	8.05	Overlapping doublet	8.52	131.3

**Fig. 6:**
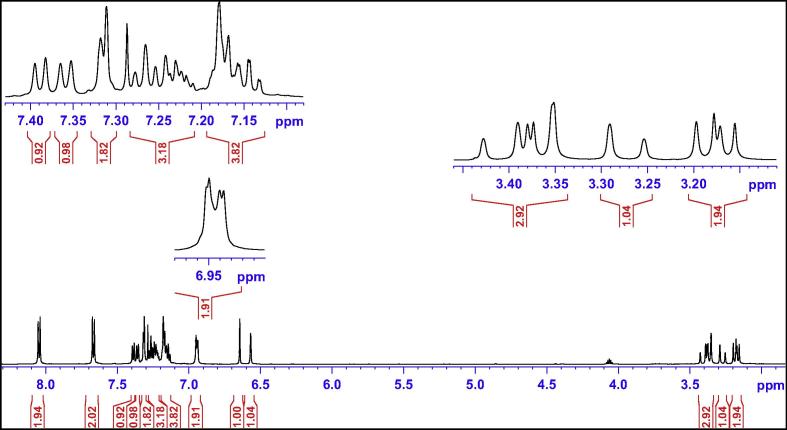
^1^H NMR spectrum of compound (**4**).

**Fig. 7:**
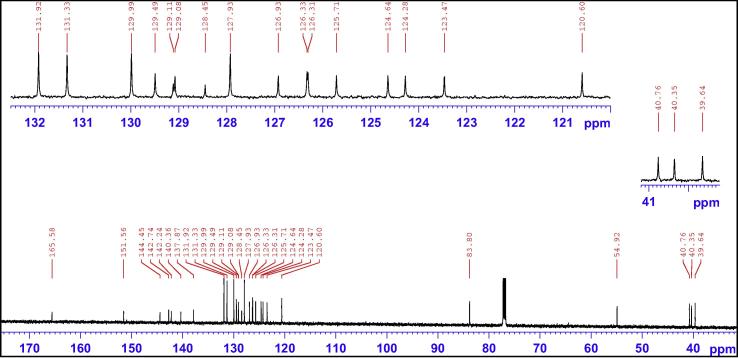
^13^C NMR spectrum of compound (**4**).

The most logical approach was to identify a proton in ^1^H NMR spectrum (Fig. 6) (or a carbon in ^13^C NMR spectrum in Fig. 7) that displayed a well-defined chemical shift value and subsequently to employ HSQC spectra to identify its corresponding carbon (or proton). The most distinct peak in the ^13^C NMR spectrum (the starting point) was that of the benzylic tertiary carbon (C2) linking to the ester group at 83.8 ppm. From HSQC spectrum, the corresponding proton (H2) was seen at 6.64 ppm as a sharp singlet. The double bond proton (H25) and carbon (C25) was subsequently identified at 6.57 ppm as a singlet in ^1^H spectrum and at 129.5 ppm in ^13^C NMR spectrum.

**Fig. 8:**
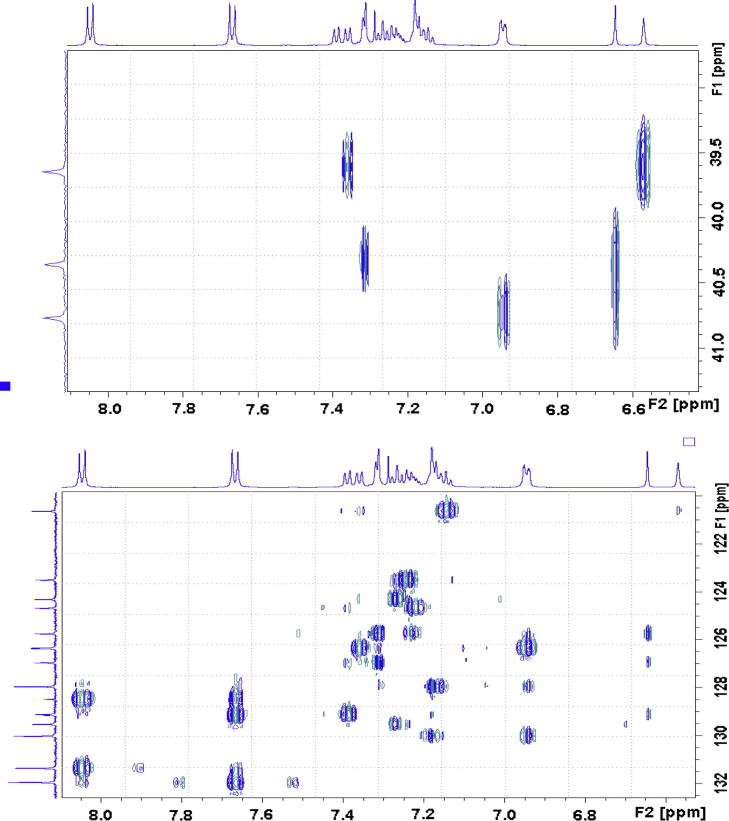
Aliphatic (upper) and aromatic (lower) regions of HMBC NMR spectrum of compound (**4**).

The three-bond couplings of H25 to a secondary carbon, C18, resonating at 39.6 ppm and a tertiary carbon, C23, resonating at 120.6 ppm were observed in HMBC spectrum (Fig. 8).

**Fig. 9:**
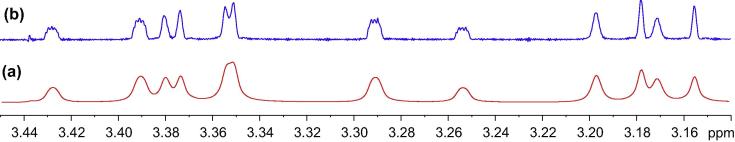
(a) Expansion of aliphatic region of ^1^H NMR spectrum of compound (**4**); (b) spectral deconvolution of aliphatic region of compound (**4**).

**Fig. 10:**
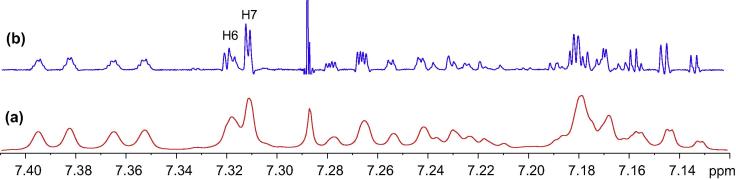
(a) Expansion of aromatic region of ^1^H NMR spectrum of compound (**4**); (b) spectral deconvolution of aromatic region of compound (**4**).

Unfortunately, the signals in the ^1^H NMR spectrum were poorly resolved and broad in the aliphatic region. HSQC correlation therefore could not accurately identify the corresponding methylene protons (Fig. 9a). To achieve some form of measurement noise reduction, and to more accurately distinguish the different chemical constituents, spectral deconvolution technique was employed by using a Gaussian multi-function (Fig. 9b). The signals in both aliphatic and aromatic regions (Fig. 10) were sharpened and well separated.

**Fig. 11:**
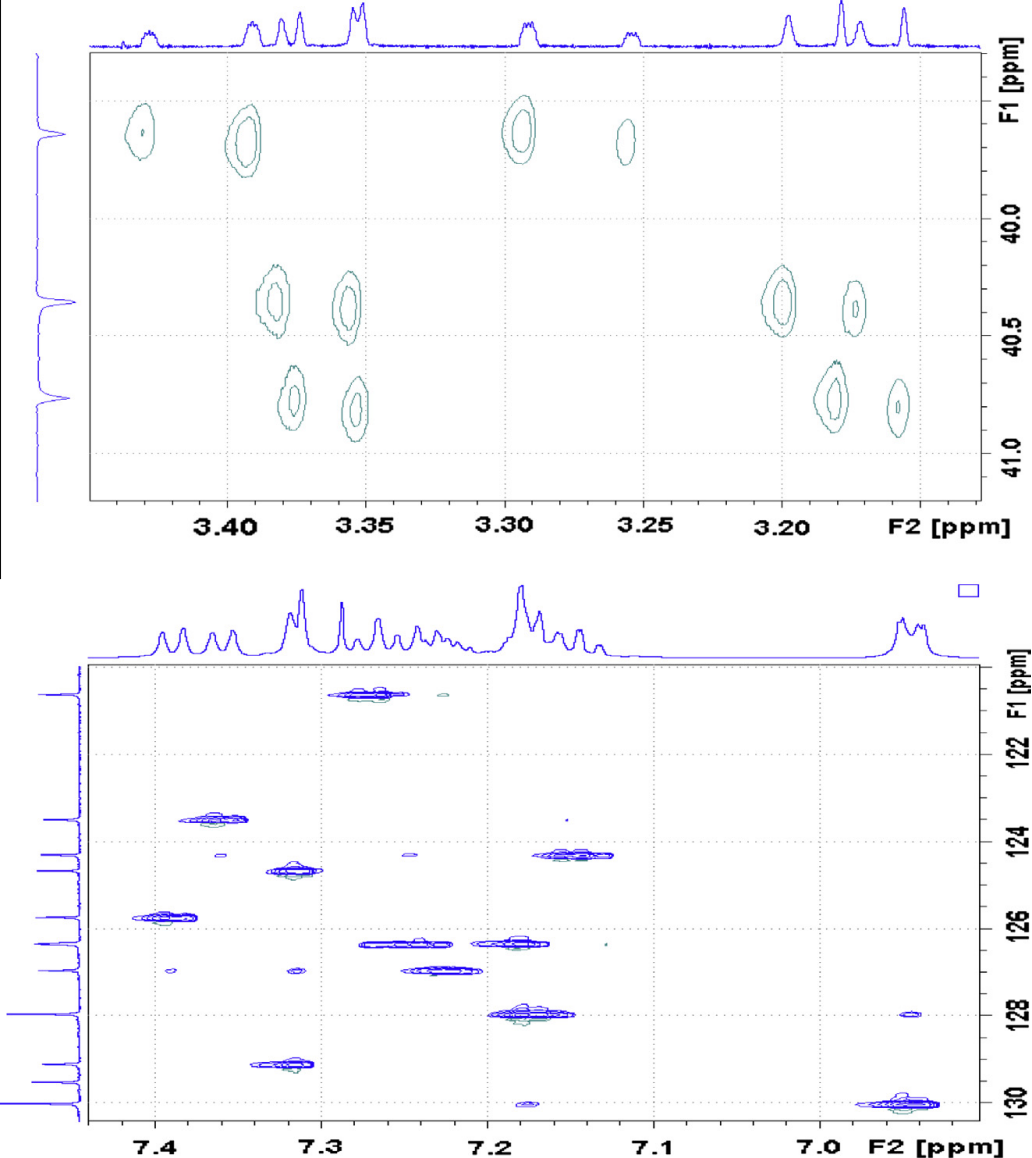
Aliphatic (upper) and aromatic (lower) regions of HSQC NMR spectrum of compound (**4**).

With an aid of HSQC experiment, two methylene protons at H18, were located at 3.27 and 3.41 ppm as two doublets (*J* = 22.52 Hz) (Fig. 11), while H23 at 7.27 ppm as a triplet (*J* = 7.16 Hz). C20 was identified at 123.5 ppm *via* three-bond coupling with H18 in HMBC spectrum. Its corresponding proton, H20, resonated at 7.36 ppm as an overlapping doublet (*J* = 7.60 Hz). Two weak contours representing the couplings of H23 to C22 at 126.33 ppm and H22 to C21 at 124.3 ppm were evident in HSQC spectrum. H5 and H22 offered a complex overlapped spectra pattern in the region from 7.21 to 7.24 ppm, which prohibited reliable measurements of coupling constants. However, it was believed that signals centered at 7.23 and 7.24 ppm were represented as H5 and H22 respectively. H21 at 7.15 ppm appeared as a double triplet (*J*_1_ = 1.23 Hz and *J*_2_ = 7.29 Hz), resulting from the direct couplings to H22 and H20, as well as meta-coupling to H23.

**Fig. 12:**
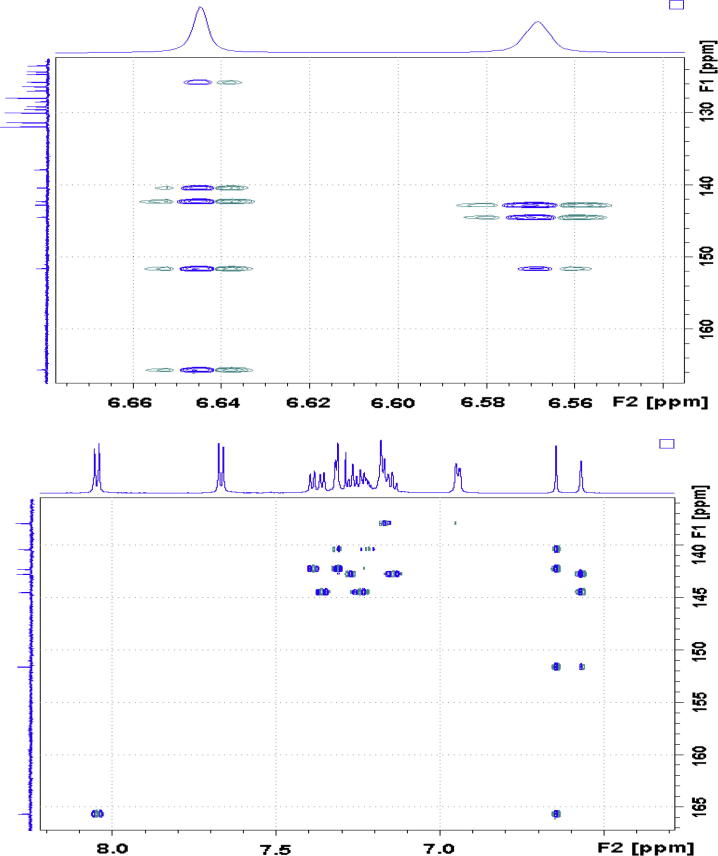
Expansions of aromatic regions of HMBC NMR spectrum of compound (**4**).

**Fig. 13:**
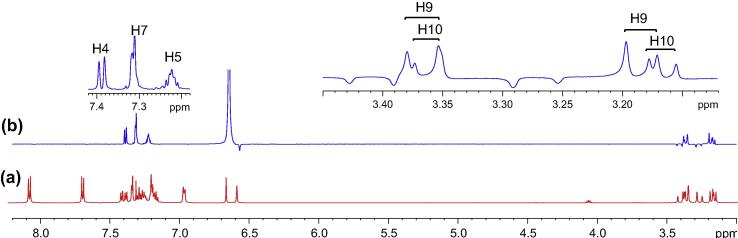
(a) ^1^H NMR spectrum of compound (**4**); (b) 1D selective TOCSY NMR spectrum of compound (**4**) with o1p 6.64 ppm.

Correlation of H2 to a tertiary carbon, C4, at 125.7 ppm and a carbonyl carbon, C27, at most downfield at 165.6 ppm were evident from HMBC spectrum (Fig. 12). From HSQC spectrum (Fig. 11), it was clear that the H4 doublet at 7.39 ppm correlated with two carbon atoms at 125.7 and 126.9 ppm with unequal coupling intensities, suggesting the stronger and weaker contours to be C4 (at 125.7 ppm) and C5 (at 126.9 pm) respectively. HSQC analysis further indicated H5 with a resonance position of being centered at 7.23 ppm. A contour in HMBC spectrum indicative of correction of C5 to H7 (over three bonds) and H6 (over two bonds) demonstrated the overlapped signal in the region from 7.30 to 7.33 ppm was attributed to H6 and H7 (Fig. 8). Application of signal deconvolution resulted in the observations of an apparent doublet (at 7.311 ppm) and unwell-solved triplet (at 7.319 ppm), in which were accounted for H7 and H6 respectively (Fig. 10). Three-bond coupling existed between H7 and C9, which was found to resonate at 40.4 ppm in HMBC spectrum, while its corresponding proton, H9, presented at 3.19 and 3.371 ppm as two doublets (*J* = 15.42 Hz). These assignments were in agreement with the 1D selective TOCSY spectral data (Fig. 13).

**Fig. 14:**
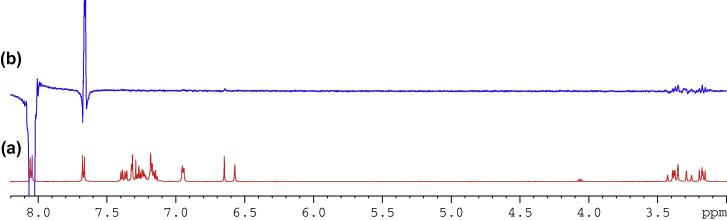
(a) ^1^H NMR spectrum of compound (**4**); (b) 1D selective NOE spectrum of compound (**4**) following irradiation of signal at 8.05 ppm.

HMBC spectrum illustrated correlations of C27 to a doublet (*J* = 8.52 Hz), H34 and H30, locating at most downfield 8.05 ppm *via* three-bond coupling (Fig. 12). Analysis of HSQC suggested correlations of H34 and C30 to an aromatic tertiary carbon at 131.3 ppm. The doublet at 8.05 ppm therefore represented two equivalent protons attached to C34 and C30. C32 was easily located at 128.5 ppm in HMBC spectrum over three-bond coupling to H34 and H30. Due to the electronegativity of bromine atom, the doublet (*J* = 8.68 Hz) at 7.67 ppm was believed to be account for H33 and H31, while their corresponding carbons, C33 and C31 had an overlapped chemical shift of 131.9 ppm. 1D selective NOE spectral data confirmed such assignments. Strong correlations between H34&H30 and H33&H31 were clearly observed (Fig. 14).

**Fig. 15:**
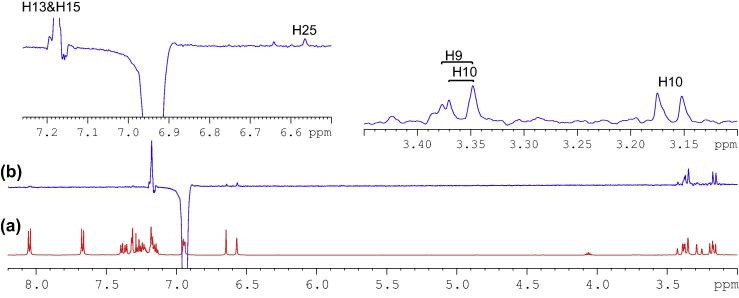
(a) ^1^H NMR spectrum of compound (**4**); (b) 1D selective NOE spectrum following irradiation of signal at 6.94 ppm.

Having identified methylene protons of H9 and H18, the last pair of methylene protons, H10, was straightforwardly confirmed at 3.17 and 3.366 ppm as two doublets (*J* = 13.56 Hz), while the corresponding carbon, C10, at 40.8 ppm by HSQC analysis. Correlation of C10 *via* three-bond coupling to a multiplet centered at 6.94 ppm identified H12 and H16. HSQC spectrum presented the strong contour of H12 and H16 to aromatic carbon C12 and C16 at 130.0 ppm, and the weaker contour at 127.9 ppm representing as C13 and C15 ppm. Their protons, H13 and H15 were located in HSQC spectrum as an overlapped signal, multiplet, with a chemical shift range of 7.16–7.19 ppm. The last remaining unassigned tertiary carbon, C14, was identified at 126.31 ppm. From HSQC analysis, the signal of H14 was also overlapped with those of H13 and H15, showing as a complex multiplet between 7.16 and 7.19 ppm. Very strong through-space correlations of H12&H16 with H13&H15 and H10 were seen, with the detections of medium correlation with one proton at C9 and weak correlation with H25 in 1D selective NOE spectrum (Fig. 15). HMBC experiment proved to be a very powerful technique in determining the exact positions of all remaining aromatic quaternary carbons.

Having assigned all proton and carbon signals, the relative chemical structure of compound (**4**) required confirmation. To assess the molecular configuration, a 3D physical molecular model was employed. It was expected that the stereochemical orientations of the benzylic proton, H2, and the indene moiety, C17 to C18, should be the identical, both either coming out of or pointing away from the plane. Such arrangement was conveniently and successfully confirmed following inspections of both 1D and 2D NMR NOSEY experiments.

**Fig. 16:**
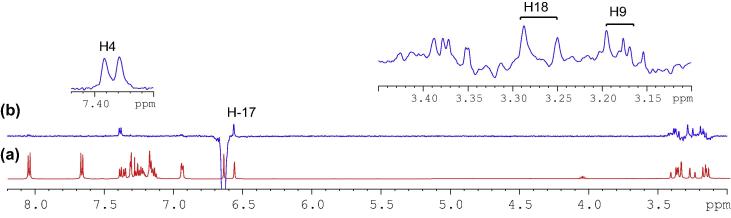
(a) ^1^H NMR spectrum of compound (**4**); (b) 1D selective NOE spectrum following irradiation of signal at 6.64 ppm.

**Fig. 17:**
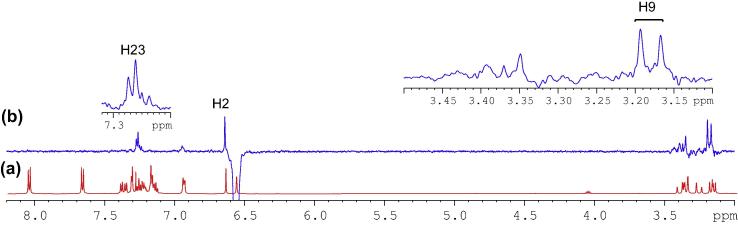
(a) ^1^H NMR spectrum of compound (**4**); (b) 1D selective NOE NMR spectrum following irradiation of signal at 6.57 ppm.

**Fig. 18:**
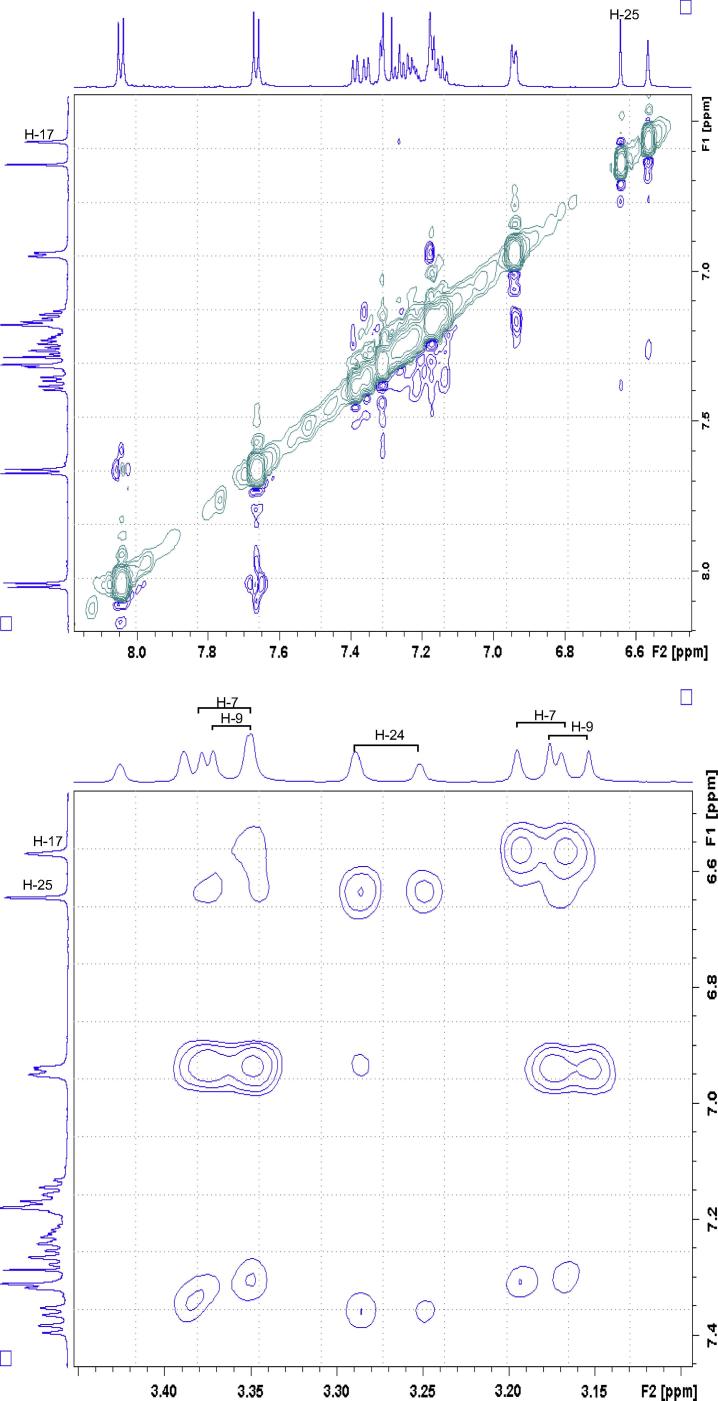
Aromatic (upper) and aliphatic (lower) region of 2D NOE NMR spectrum of compound (**4**).

In Fig. 16, Fig. 17, Fig. 18, strong correlations indicating through-space coupling among the protons at positions of H2, H25, H18 (one proton involved only) and H9 (one proton involved only) established that both groups (benzylic H2 and indene ring) were situated in the same orientation.

## Conclusion

The molecular structure and the absolute configuration of the pure single enantiomer (**2**), (1*S*,2*S*)-2-benzyl-2,3-dihydro-2-(1H-inden-2-yl)-1H-inden-1-ol, was determined based on the studies carried out on its corresponding brominated derivate (**4**), (1*S*,2*S*)-2-benzyl-2,3-dihydro-2-(1H-inden-2-yl)-1H-inden-1-yl 4-bromobenzoate. The insertion of a heavy atom, like bromine in our case, proved to be very useful during the process of crystallisation and crystallographic analysis. Various 1D (1H, 13C, selective TOCSY, ROSEY and NOESY) and 2D (COSY, HSQC, HMBC and NOESY) NMR spectroscopic experiments were carried out successfully for the structural elucidation process. We found HMBC NMR spectral data extremely useful in the course of compound characterisation. NMR TOCSY and NOESY experiments also allowed us to determine the relative configuration of enantiomer (**4**), in which both benzylic protons at H25 position and the indene functionality of the molecule had the same chemical orientations. The absolute configuration (as *S*, *S*) at C1 and C2 positions was established by crystallographic analysis, the Flack parameter was determined to be 0.010(14). The XPRD analysis confirmed the pattern of compound (**4**) generated from the single crystal X-ray was in agreement with the experimental XPRD pattern of the bulk materials. H–H and H–C intermolecular interactions related to indene moiety play predominant role in stabilization of crystal lattice of compound (**4**) molecular structure.

## Supplementary materials

CCDC 1029109 contains the supplementary crystallographic data for this paper. These data can be obtained free of charge via http://www.ccdc.cam.ac.uk/conts/retrieving.html (or from the Cambridge Crystallographic Data Centre, 12, Union Road, Cambridge CB2 1EZ, UK; fax: +44 1223 336033).

## Footnotes

1 For interpretation of color in Fig. 2, the reader is referred to the web version of this article.

## References

[1] Bai H., Chen X., Zhang L., Dou X. (2012). BMC Gastroenterol..

[2] Scheper M.A., Nikitakis N.G., Chaisuparat R., Montaner S., Sauk J.J. (2007). Neoplasia (New York, N.Y.).

[3] Shiff S.J., Qiao L., Tsai L.L., Rigas B. (1995). J. Clin. Investig..

[4] Vacca J.P., Dorsey B.D., Schleif W.A., Levin R.B., McDaniel S.L., Darke P.L., Zugay J., Quintero J.C., Blahy O.M., Roth E. (1994). Proc. Natl. Acad. Sci. USA.

[5] Moyer T.P., Temesgen Z., Enger R., Estes L., Charlson J., Oliver L., Wright A. (1999). Clin. Chem..

[6] Sheridan H., Lemon S., Frankish N., McArdle P., Higgins T., James J.P., Bhandari P. (1990). Eur. J. Med. Chem..

[7] Sheridan H., Frankish N., Farrell R. (1999). Planta Med..

[8] Sheridan H., Frankish N., Farrell R. (1999). Eur. J. Med. Chem..

[9] Sheridan H., Butterly S., Walsh J.J., Cogan C., Jordan M., Nolan O., Frankish N. (2008). Bioorg. Med. Chem..

[10] Sheridan H., Walsh J.J., Cogan C., Jordan M., McCabe T., Passante E., Frankish N.H. (2009). Bioorg. Med. Chem. Lett..

[11] Sheridan H., Walsh J.J., Jordan M., Cogan C., Frankish N. (2009). Eur. J. Med. Chem..

[12] Farrell R., Kelleher F., Sheridan H. (1996). J. Nat. Prod..

[13] Frankish N., Farrell R., Sheridan H. (2004). J. Pharm. Pharmacol..

[14] Frankish N., Sheridan H. (2012). J. Med. Chem..

[15] Sheldrick G.M. (2008). Acta Crystallogr..

[16] Blessing R. (1995). Acta Crystallogr. Section A.

[17] Farrugia L. (1997). J. Appl. Crystallogr..

[18] Spackman M.A., Jayatilaka D. (2009). CrystEngComm.

[19] McKinnon J.J., Jayatilaka D., Spackman M.A. (2007). Chem. Commun..

[20] Paluch K.J., Tajber L., Elcoate C.J., Corrigan O.I., Lawrence S.E., Healy A.M. (2011). J. Pharm. Sci..

[21] Paluch K.J., McCabe T., Muller-Bunz H., Corrigan O.I., Healy A.M., Tajber L. (2013). Mol. Pharm..

[22] Spackman M.A., McKinnon J.J. (2002). CrystEngComm.

[23] McKinnon J.J., Spackman M.A., Mitchell A.S. (2004). Acta Crystallogr. Section B.

